# Neonatal cholestasis as the onset symptom of McCune–Albright syndrome: case reports and a literature review

**DOI:** 10.3389/fped.2023.1275162

**Published:** 2023-10-11

**Authors:** Weiyuan Fang, Yanhui Zhang, Lian Chen, Xinbao Xie

**Affiliations:** ^1^ Pediatric Liver Center, Children’s Hospital of Fudan University, Shanghai, China; ^2^ Infectious Disease Department, Qingdao Women and Children’s Hospital, Qingdao, China; ^3^ Department of Pathology, Children’s Hospital of Fudan University, Shanghai, China

**Keywords:** McCune–Albright syndrome (MAS), neonatal cholestasis, GNAS, liver histology, liver

## Abstract

**Aim:**

This study aimed to summarize and show the characteristics and evolutionary process of neonatal cholestasis caused by McCune–Albright syndrome (MAS), as neonatal cholestasis may be the initial manifestation of MAS before other classic clinical features appear.

**Methods:**

The clinical characteristics, treatment methods, and outcomes of three neonatal cholestasis cases caused by MAS in our center were retrospectively studied. In addition, all the reported cases of MAS combined with cholestasis were reviewed and summarized to show the cholestatic features in them.

**Results:**

We have confirmed three MAS cases in our center, presenting onset symptoms of jaundice, pale stool, and neonatal cholestasis soon after birth. The cholestasis subsided spontaneously at around the sixth month. The literature review showed that the levels of total bilirubin, conjugated bilirubin, ALT, AST, and GGT in neonatal MAS cholestasis cases were 207 μmol/L (range 65–445 μmol/L), 162 μmol/L (range 46–412 μmol/L), 821 U/L (range 85–3,597 U/L), 532 U/L (range 127–3,633 U/L), and 244 U/L (range 79–3,800 U/L), respectively. Liver histology showed canalicular and hepatocellular cholestasis, giant hepatic cell transformation, and bile paucity. Extrahepatic manifestations such as café-au-lait pigmented skin lesions, Cushing's syndrome, hyperthyroidism, renal tubular dysfunction, and skeletal abnormalities could occur simultaneously when jaundice occurred. GNAS mutations had a high positive rate (83.3%–100%) in liver tissue with cholestasis. Neonatal cholestasis caused by MAS could be self-resolved, but hepatic lesions persist and have malignant potential.

**Conclusion:**

MAS can be one of the causes of neonatal cholestasis, which may be the first manifestation of the disease. Extrahepatic coexisting symptoms of MAS and liver histology can help to distinguish MAS from other etiology of cholestasis. Detecting GNAS mutations in liver tissue may shorten diagnostic time and is of particular interest in the partial and atypical forms of MAS with neonatal cholestasis. Neonatal cholestasis in children with MAS can self-resolve, but liver dysfunction and malignant lesions persist.

## Introduction

McCune–Albright syndrome (MAS) is a rare disease involving multiple organs and systems, characterized by café-au-lait pigmented skin lesions, polyostotic fibrous dysplasia (FD), and endocrine dysfunctions. It is caused by somatic activating mutations in the gene encoding guanine-nucleotide-binding protein (G protein) α-subunit (Gsα). The somatic mutations can occur in different tissues and at different developmental stages, leading to varied and complex clinical manifestations, often involving the bone, endocrine, heart, kidney, and liver. Neonatal cholestasis may be the first attack symptom of MAS before other classic clinical characteristics appear. In this study, we report for the first time three cases of MAS beginning with infantile cholestasis in China and combine them with similar cases to show the characteristics and evolutionary process of cholestasis caused by MAS, which aids the understanding of infant hepatic phenotypes in MAS.

## Methods

We retrospectively reviewed MAS children identified from the hepatology database of the pediatric liver center at Children's Hospital of Fudan University from 1 January 2016 to 1 July 2023. At least two of the cardinal clinical features, namely, monostotic/polyostotic fibrous dysplasia, café-au-lait skin pigmentation, and hyperfunctioning endocrinopathies, emerged to set the diagnosis of MAS (1). Hyperfunctioning endocrinopathies include gonadotropin-independent precocious puberty, growth hormone excess, non-autoimmune hyperthyroidism, hyperprolactinemia, or neonatal hypercortisolism.

For the literature review, “McCune–Albright syndrome” was used as the keyword to search the literature in the PubMed database up to July 2023. The MAS cases combined with cholestasis were selected for further analysis and review.

## Case reports

Three patients were identified as having MAS: two boys and one girl. All of them were Chinese and born to non-consanguineous healthy parents. Case 1 was a term neonate with a birth weight of 2.6 kg and normal weight gain after birth. Case 2 was a term neonate with a birth weight of 3.37 kg but had a faltering growth at the time of referral (3.7 kg at the age of 51 days). Case 3 was born at 36 weeks of pregnancy, with a birth weight of 2.4 kg and normal weight gain after birth.

All patients exhibited jaundice and pale stool a few days after birth and were transferred to our liver center due to persistent and unexplained jaundice for more than 1 month. The median age at the time of referral was 48 days (range 34–69 days), the median time from presentation to diagnosis was 21 months (range 6–46 months), and the median follow-up time was 5 years (range 4–6 years).

They all had no particular facial features except for Case 1, who had a positive posterior corneal embryonic ring examined by an ophthalmologist. All patients suffered from hepatomegaly, elevated transaminase levels, and conjugated hyperbilirubinemia (Table 1). Blood routine and coagulation tests were normal. A liver ultrasound did not show special abnormalities except contracted gallbladder and hepatomegaly. Other first-line investigations conducted according to the guidelines for neonatal conjugated jaundice were also non-contributory (2).

**Table 1:** Demographics and clinical characteristics of three MAS cases presenting with neonatal cholestasis.

	Case 1	Case 2	Case 3
Sex	Female	Male	Male
Age at presentation	Days after birth	Days after birth	Days after birth
Age at referral	51 days	34 days	69 days
Age at diagnosis	6 months	46 months	21 months
Age at last visit	6 years	5 years	4 years
Hepatic manifestations at presentation			
Stool color	Pale	Pale	Pale
Hepatomegaly	Yes	Yes	Yes
TB (peak)	185 (207)	182 (182)	150 (167.8)
DB (peak)	122 (134)	98.3 (98.3)	123 (149.7)
Bile acid (peak)	113 (237)	100 (100)	81 (286)
ALT (peak)	1,322 (2,312)	448 (821)	495 (2,254)
AST (peak)	1,102 (1,742)	276 (742)	418 (1,516)
GGT (peak)	105 (522)	128 (858)	79.1 (249)
Abdominal ultrasound	Contracted gallbladder	Liver enlargement	Contracted gallbladder, liver, and spleen enlargement
HIDA	NA	Delayed bile excretion	Complete biliary obstruction
Liver histology	Giant cell transformation, cytoplasmic cholestasis, hepatocellular necrosis, inflammatory infiltration, fibrous tissue hyperplasia, separation of liver lobules, and mild bile ducts hyperplasia	NA	Giant cell transformation, cytoplasmic and canalicular cholestasis, spotty hepatic necrosis, extramedullary hematopoiesis, fibrous tissue hyperplasia, separation of liver lobules, moderate inflammatory infiltration, and mild bile ducts hyperplasia
Liver outcome	Jaundice resolved at the age of 3.5 months, ALT and AST normalized at the age of 3.5 years, and GGT remained abnormal (65 IU/L). Liver ultrasonography: normal	Jaundice resolved at the age of 4.5 months, ALT and AST normalized at the age of 4.7 years, and GGT remained abnormal (75 IU/L). Liver ultrasonography: normal	Jaundice resolved at the age of 10 months, all of ALT, AST, and GGT normalized at the age of 23 months. Liver ultrasonography: normal
Extrahepatic manifestations at presentation			
Skin (café-au-lait spots)	At birth	2 months after birth	At birth
Kidney	Ultrasound scan showed homogenous architecture without visible differentiation between parenchyma and renal sinus	Normal	Renal tubular acidosis
Echocardiography	Normal	Atrial septal defect	Normal
Endocrine system	Vaginal bleeding at the age of 6 months	Thyroid nodules at the age of 5 years	Normal
Skeleton	Fibrous dysplasia at the age of 21 months	Fibrous dysplasia at the age of 46 months, bone biopsy: fibrocartilaginous dysplasia	Fibrous dysplasia at the age of 21 months

TB, total bilirubin (reference values: 3.4–17.1 μmol/L); DB, direct bilirubin (reference values: 0–6 μmol/L); bile acid (reference values: 0–10 μmol/L); ALT, alanine aminotransferase (reference values: 7–30 U/L); AST, aspartate aminotransferase (reference values: 14–44 U/L); GGT, gamma-glutamyl transpeptidase (reference values: 9–150 U/L); NA, not available.

**Figure 1:**
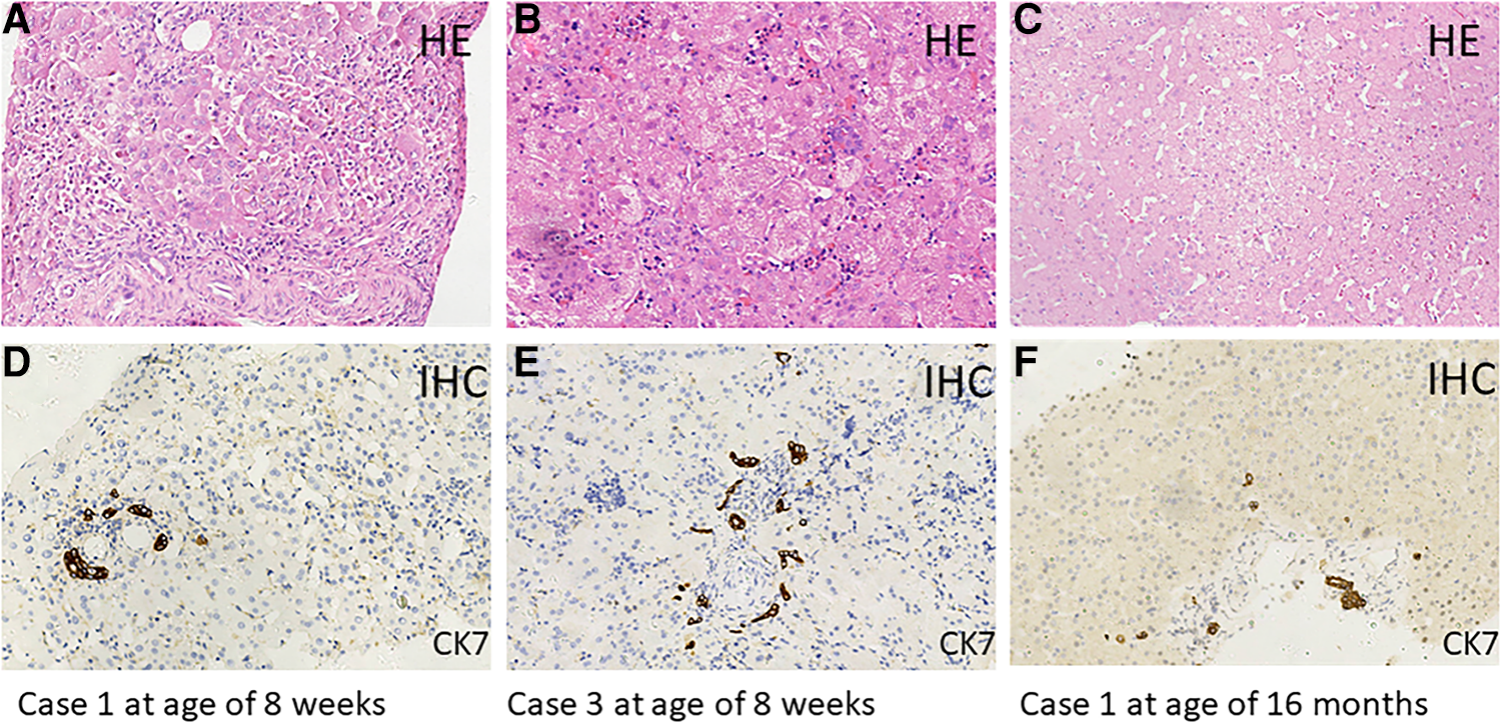
Hematoxylin–eosin (HE) staining of liver tissue sections from Case 1 (age of 8 weeks: **A**,**D**) (age of 16 months: **C**,**F**) and Case 3 (age of 8 weeks: **B**,**E**). Immunohistochemical (IHC) staining of cytokeratin 7 (CK7), which is a specific marker for bile duct epithelial cells. Original magnification, ×200.

MAS was suspected to be the cause of neonatal cholestasis in Case 1 promptly as Café-au-lait macules were discovered at birth. A skeletal survey was performed, and a complete x-ray film showed suspicious osteopenia in the middle and lower parts of the left tibia; however, it did not reveal findings consistent with FD. Bone scintigraphy in Case 1 was normal. A liver biopsy was performed for her at the age of 2 months, and the results showed ballooning degeneration of hepatocytes with multinucleation and giant cell transformation. Cholestasis in the cytoplasm of liver cells, necrosis of some liver cells with moderate inflammation, and obvious inflammation in the portal area were also observed. In addition, fibrous tissue hyperplasia, separation of lobules of the liver, mild hyperplasia of the bile duct, and extramedullary hematopoiesis were found (Figure 1). She was confirmed as MAS at the age of 6 months when she presented vaginal bleeding and breast tissue development.

Biliary atresia was excluded by hepatobiliary scintigraphy in Case 2, which manifested as poor uptake and delayed excretion at the first admission. Afterward, he was lost to follow-up. Café-au-lait macules gradually appeared after 2 months of age, later than the onset of cholestasis. MAS was still not identified at the local primary hospital where his parents took him when he presented abnormal posture and gait at 1.5 years old and when he developed several fractures at around 2 years old. MAS was not diagnosed until he came to our hospital at the age of 3 years and 10 months again due to recurring fractures. Typical skeletal radiographic abnormalities of polyostotic fibrous dysplasia were found, and the destructed bone tissue obtained during orthopedic surgery showed fibrocartilaginous dysplasia with abundant chondrocytes and local atypical hyperplasia, consistent with the characteristics of MAS.

Case 3 also presented Café-au-lait macules at birth; therefore, MAS was suspected. However, in this 70-day-old male infant with pale stool and cholestasis, the hepatobiliary iminodiacetic acid (HIDA) scan showed poor uptake and blocked excretion. A laparoscope exploration with intraoperative cholangiography was performed for him at the age of 8 weeks, showing that the extrahepatic biliary tree was patent and normal. Liver tissues from laparoscope exploratory operation showed ballooning degeneration of hepatocytes with multinucleation and giant cell transformation, cytoplasmic and canalicular cholestasis, spotty hepatocellular necrosis with moderate inflammation, fibrous tissue hyperplasia in the portal tract area, partial separation of liver lobules with moderate inflammation, and mild bile duct hyperplasia. In addition, much extramedullary hematopoiesis was observed (Figure 1). MAS was confirmed at the age of 21 months when typical skeletal radiographic abnormalities of polyostotic fibrous dysplasia were found during subsequent regular follow-ups.

At the same time, other MAS comorbidities were also assessed (Table 1). None had a heart murmur, but echocardiography demonstrated a secundum atrial septal defect in Case 2. Case 3 demonstrated renal involvement, manifested as proximal renal tubular acidosis.

In the three MAS patients with neonatal cholestasis, both bilirubin and transaminase levels peaked at a median period of 67 days (range 39–100 days) (Table 1). All infants were treated with ursodeoxycholic acid, fat-soluble vitamins, and nutritional supplementation with a medium-chain triglyceride. Cholestasis resolved at a median time of 6 months (range 3.5–10 months). Transaminases had been fluctuating for a median period of 40 months (range 23–56 months), but they finally returned to normal. In addition, no liver lesion was detected on routine abdominal ultrasound scans. However, the GGT levels remained persistently abnormal in Cases 1 and2, ranging from 60 to 110 U/L. Case 1 underwent a second liver biopsy at the age of 16 months due to persistently abnormal liver function, and the results showed hydropic degeneration of hepatocytes along with only mild fibrosis and lymphocytic infiltration (Figure 1).

All Café-au-lait macules followed Blaschko's lines and were distributed in multiple parts of the skin including the face, thoracoabdominal and dorsal regions, arms, and legs. No more Café-au-lait macules continued to appear after 2 months. All children received bisphosphonate therapy as typical skeletal radiographic abnormalities of polyostotic fibrous dysplasia were found in them around the age of 1.5 years. Recurrent femur fractures happened in Case 2, whereas exercise tolerance in Cases 1 and 3 was also limited by the bone lesions. Case 1 received aromatase inhibitor therapy after she presented vaginal bleeding and breast tissue development. No gonadotropin-independent precocious puberty was ever found in the male patients. Except for Case 2, in which complex thyroid nodules (solid and cystic) were found at the age of 5 years, no endocrine dysfunctions related to the thyroid, adrenal, and pituitary glands were found.

The results of the whole exon sequencing of blood samples from all three patients were negative. Moreover, their parents were reluctant to allow us to perform further investigations on the liver or bone biopsy tissues of their children.

## Discussion

McCune–Albright syndrome is caused by GNAS gene mutations on somatic cells; therefore, the phenotypes of an individual may vary widely, depending on the embryonic stage during which the mutation occurs in the affected tissues and the expression levels of Gsα in the affected tissues. The affected tissues that have been reported include the endocrine glands, skin, skeleton, kidney, heart, pancreas, and liver. Hepatobiliary dysfunction in MAS is relatively rare, with a frequency of 5%–10% (3, 4). The occurrence and severity of the hepatic phenotype depend on the number and location of the mutant cells (5, 6). The known hepatobiliary manifestations of MAS include neonatal cholestasis hepatitis, hepatic adenoma, focal nodular hyperplasia, and hepatic tumor (1, 4, 7–9). There were 12 studies (5–7, 10–18) involving 16 patients with cholestasis caused by MAS, and the detailed information had been reported and could be found in the PubMed database. The features of neonatal cholestasis in the 19 MAS patients, including the 16 MAS patients reported by others and the three MAS patients mentioned above by us, are summarized in Supplementary Table S1.

**Figure 2:**
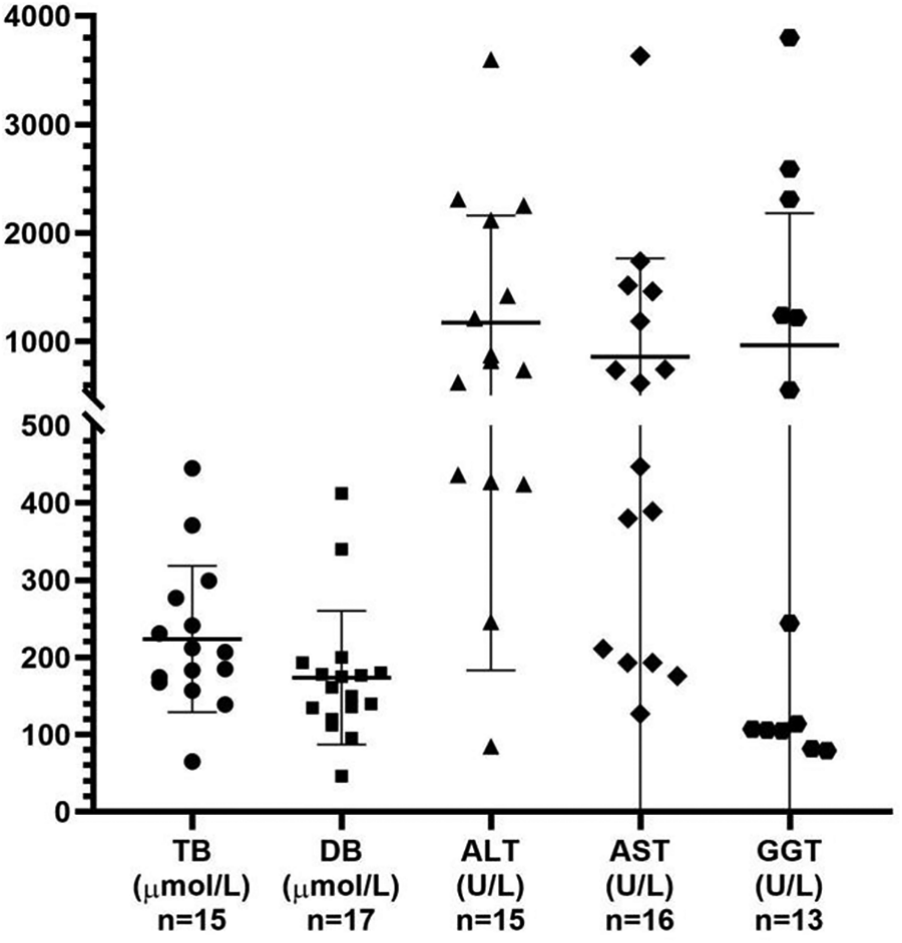
Analysis of the liver function test results obtained from the reviewed 19 patients with MAS presenting with cholestasis. TB, total bilirubin; DB, direct bilirubin; ALT, alanine aminotransferase; AST, aspartate aminotransferase; GGT, gamma-glutamyl transpeptidase.

The 10 male patients vs. nine female patients showed no significant gender difference between MAS patients with cholestasis. Only three patients were premature, while the majority were delivered at term, of which 7/16 of the patients had low birth weight. Among the 19 patients, 17 patients presented jaundice during the neonatal period, one patient showed jaundice at the age of 3 months with increased liver enzymes 2 months earlier, one patient was confirmed as cholestasis due to an increased GGT level but without jaundice, seven patients showed pale stool, and a similar proportion had hepatomegaly. The levels of total bilirubin, conjugated bilirubin, ALT, AST, and GGT in these patients with cholestasis were 207 μmol/L (range 65–445 μmol/L), 162 μmol/L (range 46–412 μmol/L), 821 U/L (range 85–3,597 U/L), 532 U/L (range 127–3,633 U/L), and 244 U/L (range 79–3,800 U/L), respectively (Figure 2). Abdominal ultrasonography generally displayed an abnormally small and contracted gallbladder, with a thick gallbladder wall and echogenic material, and no other special signs in most cases. However, choledochal cysts and atresia were found by ultrasonography in one patient, and bile duct obstruction and partial obstruction were often found by the HIDA scan.

These above characteristics of cholestasis caused by MAS, including pale stool, high GGT level, and bile duct obstruction, were similar to those observed in biliary atresia, and 6/19 of the patients did indeed undergo intraoperative cholangiography. In fact, the main liver histopathological manifestations of these MAS patients with cholestasis, including canalicular and hepatocellular cholestasis, giant hepatic cell transformation, a reduced number of intrahepatic bile ducts or bile duct paucity, and extramedullary hematopoiesis, were totally different from those of biliary atresia and could be easily distinguished from the multivariate model for diagnosing biliary atresia that includes bile duct hyperplasia, portal fibrosis, and absence of non-sinusoidal fibrosis. It should be noticed that two of our patients showed bile duct hyperplasia or mild hyperplasia rather than bile duct paucity, which was still significantly different from the classical bile duct hyperplasia in biliary atresia (Figure 1). However, the mechanisms of cholestasis, bile duct paucity, and hyperplasia in MAS remained not fully understood (15).

As both MAS and Alagille syndrome (ALGS) may involve neonatal cholestasis, heart murmur or peripheral pulmonary artery stenosis (3/19 of the MAS patients), renal tubular dysfunction (Supplementary Table S1, 5/19 of the MAS patients), skeletal abnormalities, and intrahepatic bile duct paucity in liver biopsy, it is difficult to distinguish MAS from ALGS based on the above clinical symptoms and pathological features. Other extrahepatic specific symptoms coexisting with infantile cholestasis could help to distinguish from each other, such as facial features, corneal embryonic ring or butterfly vertebrae in ALGS, and café au lait macules or endocrine hyperfunction in MAS. However, only 52.5% (10/19) of the patients were found with café au lait macules, 26.3% (5/19) with Cushing's syndrome (26.3%, 5/19), and 11% (2/19) with neonatal hyperthyroidism during the same period when the cholestasis presented in the neonatal period in MAS patients, while other typical manifestations of MAS appeared later, such as bone destruction found at least 6 months later and peripheral precocious puberty developing between 6 months and approximately 7 years (Supplementary Table S2). At this moment, gene sequencing or copy number variation analysis of JAG1/NOTCH2 genes in blood samples could help in different diagnoses; however, 3.2% of the patients who meet the clinical criteria for ALGS diagnosis cannot be identified as pathogenic variants (19).

Only 21% of the DNAs extracted from peripheral blood lymphocytes (PBL) of MAS patients were positive for GNAS gene mutations due to the somatic mosaic nature of MAS (4). However, approximately 90% of the affected tissues (e.g., ovary, bone, and adrenal gland) were positive for GNAS mutations, except for café au lait macules, which only showed 27% of positive mutations (4). It was likely due to the low proportion of melanocytes, which are the cells that may mutate in skin tissue. Although negative GNAS mutation (16.6%, 1/6) had been reported in liver tissues from other MAS patients with cholestasis (4) (these MAS patients were not included in our review due to lack of detailed information about cholestasis), 100% (10/10) of liver tissues from the studied MAS infants with cholestasis were retrospectively detected with positive GNAS mutations. Based on the high positive rate of GNAS mutations in affected liver tissues and the later development of classic clinical characteristics, GNAS sequencing in liver tissues from the suspected MAS patients with cholestasis may greatly shorten the duration time from presentation to diagnosis when neonatal cholestasis is the first attack symptom without other classic clinical characteristics occurring at the same time. In our patients, GNAS mutation detection was not done in liver tissues when GNAS sequencing was negative for all PBLs, which was a limitation of our study.

In most cases, the neonatal cholestasis of MAS resolves spontaneously in about 6–12 months, although a few patients are treated with ursodeoxycholic acid and some are not. One patient (15) received a liver transplant at the age of 10 months under the setting of profound cholestasis, malnutrition, recurrent respiratory infections, and recurrent femur fractures. After liver transplantation, these comorbidities in this patient were significantly resolved or improved, except for femur fractures, which were thought to be caused by fibrous dysplasia at the final diagnosis. Four MAS patients (5, 10, 16) with cholestasis died, and all of them were associated with Cushing's syndrome. The mortality of MAS with Cushing's syndrome had been identified as 20% (6/30), and the comorbid heart and liver diseases were considered as poor prognostic markers in the same cohort as well (20).

However, liver dysfunction may persist following the amelioration of their neonatal cholestasis, and a mild-to-moderate increase in active transaminases or an isolated increase in GGT level was detected in 77% (10/13) of MAS patients. Nonetheless, six patients were given a second liver puncture biopsy between 6 months and 9 years old on account of a sustained increase in liver enzymes. Four out of six patients showed normal or approximately normal histology of the hepatic cell and bile ducts, compared to the obvious abnormality under neonatal cholestasis. Liver explant from patients (15) showed severe intrahepatic cholestasis, focal bile canalicular plugs, mild-to-moderate focal periportal, and sinusoidal fibrosis at the age of 10 months. Patients (5) showed chronic cholestasis and progressive fibrosis in liver biopsies at the ages 21 and 24 months, but upon death at 3 years and 10 months, autopsy revealed extensive focal nodular hyperplasia, bridging fibrosis, chronic cholestasis, and bile duct paucity in an abnormally large liver. Subsequent studies further demonstrated that hepatic lesions in MAS may continue to develop and even exhibit malignant potential in children, such as hepatocellular adenoma, hepatocellular carcinoma, and hepatoblastoma. Among the 19 patients reviewed, one developed hepatoblastoma at the age of 5 years, and two developed progressive atypical focal nodular hyperplasia at the ages of 6 and 7 years, respectively, 1 year after the resolution of cholestasis.

## Conclusion

Neonatal cholestasis may be the initial manifestation of MAS and may occur mostly in the first month after birth; thus, MAS should be considered as part of the differential diagnosis of neonatal cholestasis. Searching café au lait macules should become a part of routine practice, and extrahepatic manifestations, endocrine system evaluation, and liver histology can also help to distinguish other etiology of cholestasis with high GGT levels, especially biliary atresia and Alagille syndrome. Detecting GNAS mutations in liver tissue may shorten diagnostic time and is of particular interest in the partial and atypical forms of MAS with neonatal cholestasis. Neonatal cholestasis caused by MAS can resolve spontaneously, but hepatic lesions persist and have malignant potential. Liver ultrasonography and AFP monitoring should be considered for the MAS patients, especially those with neonatal cholestasis or liver dysfunction, during long-term follow-ups.

## Data Availability

The original contributions presented in the study are included in the article/**Supplementary Materials**, further inquiries can be directed to the corresponding author.
