# Supplementary material for: Neonatal cholestasis as the onset symptom of McCune–Albright syndrome: case reports and a literature review
Source: Front Pediatr. 2023 Oct 11;11:1275162. doi: 10.3389/fped.2023.1275162 (PMC10598585; doi:10.3389/fped.2023.1275162)
Supplement: Supplementary file 1 [file Table1.pdf]

Supplementary Material

Weiyuan Fang, Yanhui Zhang, Lian Chen, Xinbao Xie*

*** Correspondence:** Xinbao Xie,xxb116@163.com

Supplemental table 1. Characteristics and outcomes of cholestasis presented by the 19 reviewed MAS patients.

No.	Ref.	Sex	Hepatic manifestation	simultaneous presentation with cholestasis	Liver USS/ HIDA	Liver biopsy (age of liver biopsy)	Outcome of liver involvement (age)
1	Boston B A，etc, 1994	M	jaundice at birth	Cushing’s syndrome	NA	normal hepatocytes with some fatty infiltration (7 months).	abnormal liver function (3 years old)
2	Shenker A，etc，1993	M	jaundice at birth with raised liver enzymes	Cushing’s syndrome, hyperthyroidism；cafe´-au-lai spots	NA/ normal uptake with poor excretion	canalicular and hepatocellular cholestasis (4 weeks)<br>chronic cholestasis and progressive fibrosis<br>(two biopsies at 21 and 24 months old)	Jaundice subsided at 6 months, still with abnormal liver enzyme.<br>Died at 3 years old under unexplained cardiac arrest.
3		F	raised liver enzymes in neonates; Jaundice at the age of 3 months	low birth weight, Cushing’s syndrome, hyperthyroidism, bone abnormalities	NA	canalicular cholestasis, extramedullary hematopoiesis (3 months)	died at 2 years old for undetermined reasons
4	Silva ES，	M	jaundice at birth with liver enlargement	NA	absence of intestinal contrast	giant cell transformation with mild portal inflammatory infiltrate and	Jaundice subsided at 6 months,

	etc，<br>2000					normal bile ducts (8 weeks)<br><br>normal liver histology (11 years),	mildly raised GGT level at 11 years old.
5		F	Jaundice and unpigmented stool at birth	NA	NA	typical neonatal giant cell hepatitis (4 weeks)<br><br>normal liver histology (9 years)	Jaundice subsided at 6 months,<br><br>mildly raised GGT level at 9 years old.
6	N E，<br>etc，<br>2004	F	non-icteric cholestasis at the age of 8 months with mild hepatomegaly *	cafe´-au-lai spots	NA	moderate steatosis with severe cholestasis (8 months)	normal liver function test at 6 years old.
7	Louren co R，<br>etc，<br>2015	F	Jaundice and unpigmented stool at birth	prematurity, low birth weight,<br><br>Cushing’s syndrome, cafe´-au-lai spots	choledochal cyst and apparent atresia/inco nclusive result	NA	persistent cholestasis and liver dysfunction<br><br>died for lung infection at 4 months old
8	Ikawa Y，etc<br>，<br>2016	F	Jaundice at the age of 4 weeks	NA	NA /delayed excretion	giant cell transformation,<br><br>bile thrombus in a bile canaliculus, inflammatory	NA

						infiltrates in the hepatic parenchyma,<br><br>a normal bile duct（4 weeks）	
9	Corsi A，etc，2019	F	Jaundice at birth	Cushing’s syndrome, hyperthyroidism, heart enlarged, atrial septal defect, anemia, cafe´-au-lai spots	NA	extensive portal-based ductular reaction,<br><br>absence of biliary ducts within some portal tracts,<br><br>intralobular biliary ducts at intrahepatic ectopic sites (away from the adjacent portal trat), bile plugs, and biliary metaplasia of the hepatocytes (3 months)	Resolved cholestasis,<br><br>normal liver function<br><br>Died for lung infection<br><br>and respiratory failure<br><br>at 4 months old
10	Coles N，etc，2019	F	Jaundice at the age of 2 weeks	NA	moderate hepatomegaly/ NA	significant liver damage, cytoplasmic and canalicular cholestasis,<br><br>broad areas of resolving hepatocellular necrosis,<br><br>giant cell transformation,<br><br>abundant extramedullary hematopoiesis (1 month)<br><br>severe intrahepatic cholestasis, focal bile cannalicular plugs, mild to moderate focal peri-portal	liver transplantation due to severe cholestasis at the age of 10 months

						and sinusoidal fibrosis (10 months, liver explant)	
11		F	Jaundice and unpigmented stool at the age of 4 days	renal tubular acidosis, pulmonary stenosis, cafe´-au-lai spots	Thick-walled gallbladder with<br>echogenic material / biliary obstruction	florid neonatal hepatitis with micro abscesses and bile duct paucity<br>（4 days）	Jaundice subsided at the age of 1 year with consistently increased liver enzyme,<br>hepatic focal nodular hyperplasia at 6 years old
12	Johansen L，etc，2019	M	Jaundice and unpigmented stool at the age of 20 days	renal tubular acidosis, cafe´-au-lai spots	echogenic foci within the right lobe<br>/biliary obstruction	neonatal hepatitis with micro abscesses and necrosis<br>(20 days)	Jaundice subsided at 1 year old with consistently increased liver enzyme, hepatic focal nodular hyperplasia at 7 years old
13		M	Jaundice at birth but with normal-colored stool	renal tubular acidosis, pulmonary stenosis, left ventricular hypertrophy, cafe´-au-lai spots	abnormally small and contracted<br>gallbladder/	neonatal hepatitis and severe cholestasis (12 days)	Jaundice subsided at 1 year old with consistently increased liver enzyme, hepatoblastoma at 5 years old
14	Claeys K G，	M	Jaundice at birth and	NA	NA	hypoplasia of the internal bile ducts (9 weeks)	Jaundice subsided within 1 year

	etc，2021		hepatosplenomegaly			normal bile ducts（1 year old）	
15	Satomura Y，etc，2021	M	Jaundice at birth	renal tubular acidosis, pulmonary stenosis	NA	bile duct paucity (1 month old)	Jaundice subsided at the age of 2 years, normal liver function at 4 years old,<br><br>normal liver USS
16	Haddadi M，etc，2022	M	Jaundice at 10 days after birth with increased liver enzymes	cafe´-au-lai spots	Normal/ NA	canalicular cholestasis, extramedullary hematopoietic，giant cell formation, and a significant reduction in the number of bile (40 days)	Jaundice subsided at 6 months old still with increased liver enzymes.
17	Case 1 of this study	F	Jaundice and pale stool at 3 days after birth with liver enlargement	Positive posterior corneal embryonic ring, cafe´-au-lai spots	Contracted<br><br>gallbladder/ NA	giant cell transformation, cytoplasmic cholestasis,<br><br>mild proliferated bile ducts (1st biopsy at 2 months)<br><br>hydropic degeneration of hepatocytes with lymphocytic infiltration and mild fibrosis (2nd biopsy at 16 months)	Jaundice subsided at 3.5 months old<br><br>liver enzyme normalized at the age of 3.5 years but still with abnormal GGT level,<br><br>normal liver USS.
18	Case 2 of this study	M	Jaundice and pale stool at birth with liver enlargement	Atrial septal defect	liver enlargement / delayed excretion -	NA	Jaundice subsided at the age of 4.5 months, ALT and AST normalized at 4.7 years

							old, but still with abnormal GGT level
19	Case 3 of this study	M	Jaundice and pale stool at birth with liver enlargement	renal tubular acidosis, cafe´-au-lai spots	contracted gallbladder, hepatosplenomegaly / biliary<br>obstruction	giant cell transformation，cytoplasmic and canalicular cholestasis,<br>mild bile ducts hyperplasia (2 month）	Jaundice subsided at the age of 10 months, liver enzymes including GGT normalized at the age of 21 months,<br>normal liver USS

USS: ultrasound scan, HIDA: hepatobiliary iminodiacetic acid

*, non-icteric cholestasis: increased GGT and bile acid level but without increased bilirubin level.

NA, not available.

Supplemental table2. The onset of classic triad of MAS in the 19 reviewed patients and the GNAS sequencing results of affected tissues

No.	cafe´-au-lai spots (age)	Fibrous dysplasia (age)	Endocrine hyperactivity* (age)	Samples for sequencing	mutations of GNAS
1	NA	3.3 years	3 months**	liver, adrenals***	R 201 C
2	at birth	10 months	at birth**	pituitary, adrenal, thyroid, testes, liver, left ventricle	R 201 H
3	2 months	2 months	2 months**	adrenal glands, cafe´-au-lait spots	R 201 C
4	4 months	2.5 years	NA	liver	R 201 C
5	4 years	4 years	NA	liver	R 201 H
6	at birth	6 years	6 years	liver, blood, ovarian fluid cyst	R 201 C
7	at birth	1 month	1 month**	cheek cells	R 201 H
8	several months after birth	6 months	6 months	liver	R 201 C
9	at birth	at birth	at birth**	liver, adrenal， skeletal muscle, kidney, bone	R 201 H
10	1.2 years	1.7 years	1.2 years	liver	R 201 H

11	at birth	3 years	4 months	NA	NA
12	at birth	3 years	7 years	NA	NA
13	at birth	3 years	NA	liver	NA
14	several years after birth	several years after birth	NA	cafe´-au-lait spots	R 201 H
15	NA	4 years	NA	liver, bone tissue	R 201 C
16	at birth	2 years	4.5 years	NA	NA
17	at birth	21 months	6 months	NA***	NA
18	2 months	18 months	5 years	NA***	NA
19	at birth	21 months	NA	NA***	NA

Note: NA, not available

* Endocrine hyperactivity included precocious puberty, Cushing's syndrome, thyroid disorders, excessive secretion of growth hormone, etc.

**: The time of MAS cases diagnosed with Cushing's syndrome or hyperthyroidism.

***: Cases underwent peripheral blood GNAS mutation testing with negative results.
